# Acquired factor XII deficiency in a woman with recurrent pregnancy loss: working on a differential diagnosis in a single case

**DOI:** 10.1186/1479-5876-3-43

**Published:** 2005-12-02

**Authors:** Maristella D'Uva, Ida Strina, Antonio Mollo, Antonio Ranieri, Giuseppe De Placido, Pierpaolo Di Micco

**Affiliations:** 1 Dipartimento Universitario di Scienze Ostetriche Ginecologiche e Medicina della Riproduzione, Area Funzionale di Medicina della Riproduzione ed Endoscopia Ginecologica, Università degli Studi di Napoli Federico II, via Pansini 5 Building 9, 80131, Naples, Italy; *Thrombosis Center, Istituto Clinico Humanitas "IRCCS", via Manzoni 56, 20089, Rozzano – Milano – Italy

## Abstract

**Background:**

Antiphospholipid syndrome (APS) has been often associated to RPL since 1980 and some reports in the Literature rarely described antibodies to factor XII in patients with APS.

**Case history:**

We report the case history of 34-year-old caucasian women with recurrent fetal loss and persistent prolonged activated partial thromboplastin time. Haemostatic tests revealed persistent light decrease of clotting factor XII with normal values of IgG and IgM anticardiolipin antibodies and transient positivity for lupus anticoagulant (LA). Few reports in the Literature described antibodies to factor XII in patient with antiphospholipid syndrome (APS) and transient LA. So, once other causes of RPL were excluded, the patient was diagnosed an unusual form of APS associated to antibodies to factor XII, reduced factor XII plasma levels, transient LA and prolonged activated partial thromboplastin time.

**Discussion:**

We suggest to consider also antibodies directed to clotting factors (e.g. factor XII in our case) as second step of thrombophilia screening in RPL, in particular if a persistent prolonged aPTT is present without an apparent cause.

## Background

Recurrent pregnancy loss (RPL) is one of the most common cause of sterility. An our recent study underlined the relevant role of d-dimer, as marker of hypercoagulable state, to identify thrombophilia in women affected by primary or secondary sterility [1]

In 1999 a study written by Brenner et al. identified thrombophilia as principal cause of more than 40% of women affected by RPL [2]. Further studies underlined a pathogenetic role of inherited thrombophilia in women affected by RPL. Sanson et al., in fact, reported an increased frequency of antithrombin III, protein C and protein S deficiency in women with RPL [3], while Grandone et al. found an increased incidence of factor V Leiden in women with unexplained RPL [4]. Also prothrombin A20210G gene polymorphism has been reported as possible cause of RPL in several studies [2,5,6].

Yet, also acquired thrombophilia has been associated to RPL. A study by Dossenbach et al., in fact, revealed that elevated maternal plasma levels of clotting factor VIII tend to be associated to an increased risk of RPL [7]. Moreover, several studies in the Literature are available for the association of RPL and primary or secondary antiphospholipid syndrome [8,9]. On this topic also a rare condition as acquired deficiency of clotting factor XII has been described. Braulke et al. identified for the first time a factor XII deficiency in RPL [10], but subsequently Jones et al. reported acquired factor XII deficiency in a subpopulation of women with antiphospholipid antibodies and RPL [11-13]. We here report a really interesting case report of woman affected by unexplained RPL, prolonged activated partial thrombplastin time and mild/moderate reduction of clotting factor XII.

## Case presentation

A 34-year-old Caucasian non smoking woman was referred to our Sterility Center. Her personal anamnesis revealed three early pregnancy loss within 8 and 12 week of gestation and one extrauterine pregnancy. The patient did not revealed previous thromboembolic disease (arterial or venous) nor haemorragic disorders; moreover patient was not ongoing any type of pharmacological treatment. A thorough familial anamnesis did not show a trend toward thromboembolic and/or haemorragic disease.

To understand pathophysiology of her RPL the patient performed several laboratory and instrumental tests.

A normal ovarian function and ovulation were detected by normal values of Follicle-stimulating Hormone, Luteinising Hormone, oestradiol and progesterone and by ovarian ultrasound scan (data not shown). Uterine and salpinxes malformation was excluded by hysterosalpingography and hysteroscopy (data not shown). Endocrinological diseases such as diabetes and dysthyroidism were evaluated and excluded by normal values of glycaemia, thriiodothyronine (i.e. FT3), thyroxine (i.e. FT4) and Thyroid-stimulating Hormone (data not shown). Inflammatory chronic diseases were excluded by normal values of erythro-sedimentation rate and acute phase C reactive protein and immunopathological chronic disease, such as erytematosus systemic lupus, by normal level of antinuclear antibodies (ANA), anitmithocondrial antibodies (AMA) and smooth muscle antibodies (SMA) too (data not shown); moreover patient did not suffer of chronic joint paint or fever or other related symptoms.

Yet, routine haemostatic tests showed normal value of prothrombin time, measured as International Normalised Ratio (PT INR, 1.15) and a prolonged activated thromboplastin time, measured as ratio (aPTT, 1.45) (table 1). To confirm this laboratory alteration, after 15 days a second step of haemostatic parameters were tested and confirmed normal value of PT INR and prolonged aPTT (1.46, table 1 and 2), associated to normal levels of anticardiolipin antibodies (tested by an ELISA method; IgM 1.7 U/MPL and IgG 3.9 U/GPL) (table 1 and 2), fibrinogen and clotting factors V, VIII, IX, X, XI (table1), while a reduced plasmatic level of clotting factor XII was detected (65%, table 1 and 2). So, we tested for aPTT and factor XII first degree relatives (i.e. one sister and parents) but did not find alterations (data not shown). Yet, lupus anticoagulant and antibodies to β-2-glycoprotein I were absent (table 1 and 2). Furthermore, because RPL was associated to reduced clotting inhibitors also protein C, protein S and antithrombin III were analysed and resulted in normal range (table 1) such as inherited thrombophilia associated to Factor V Leiden and prothrombin A20210G gene polymorphisms (table 1). Moreover, to confirm factor XII deficiency, after one month the patient was evaluated again and results showed again prolonged aPTT (1.48), reduced factor XII (55%). So, we tested aPTT adding to the plasma patient's a normal plasma sample with a ratio of plasma patient's to pool plasma 1/1 v/v; results revealed a partial correction of aPTT (1.30) suggesting a possible presence of an acquired clotting inhibitor and/or antiphospholipid antibodies. A new evaluation of common antiphospholipid antibodies began with normal levels of anticardiolipin antibodies (tested again with an ELISA method; IgM 1.5 and IgG 3.8) and absence of antibodies to β-2-Glycoprotein I, but a positivity for lupus anticoagulant was detected (table 2). Lupus anticoagulant was assayed according to recommendations of the International Society of Thrombosis and Haemostasis. Furthermore, at this time we tested again ANA that resulted again negative. Finally, because a transient positivity of lupus anticoagulant has been associated with factor XII deficiency in patients with antiphospholipid syndrome in rare cases [14,15], after one month the patient was evaluated one more time and in this time also antibodies to clotting factor XII were evaluated by an ELISA method. Results showed prolonged aPTT (1.44), reduced factor XII plasma levels (43%), normal range of anticardiolipin antibodies (IgM and IgG), absence of antibodies to β-2-Glycoprotein I, negativity for lupus anticoagulant, while antibodies to factor XII have been detected (table 2). So, according to our data and data available in the Literature the patient was diagnosed an unusual form of primary antiphospholipid syndrome and an antithrombotic treatment was suggested.

## Discussion

Inherited factor XII deficiency seems to be not associated to bleeding tendency if referred to major surgery [16]. However, some individual with reduction of factor XII seems to have a trend toward thrombosis [16], but pathophysiological mechanisms underlying should be better understood. Therefore, deficiency of clotting factor XII has been reported as also thrombotic risk factor and Halbmayer et al. suggested to consider factor XII deficiency in patients with recurrent thromboembolism [17]. One of the possible mechanisms could be associated to the presence of acquired clotting inhibitors eventually associated to the presence of an antiphospholipid syndrome [18]. Acquired clotting inhibitors, in fact, have been identified in several disease and frequently are associated to a thrombophilic trend [19].

Furthermore, from a clinical point of view, alteration of haemostasis with a trend toward thrombophilia has been frequently associated to RPL [2,8]. According to a multivariate analysis on the etiology of thrombophilia by Yamada et al., clotting factor XII deficiency has been found in 4.2% of women affected by RPL [20]. Another interesting study by Iinuma et al. underlined clotting factor XII activity and not its 46C/T gene polymorphism as cause of RPL in a selected population [21]. However, factor XII deficiency may be due to inherited deficiency or acquired deficiency during an acquired disease such as antiphospholipid syndrome. In this last condition, in fact, we may find an acquired factor XII deficiency because the presence of antibodies to factor XII; moreover antibodies to factor XII may be present during antiphospholipid syndrome alone or together to lupus anticoagulant [11]: antibodies to factor XII have been described, in fact, also in patients with antiphospholipid syndrome and transient lupus anticoagulant [14,15]. Moreover, Jones et al. described that antibodies to factor XII can be present in women with RPL and antiphospholipid syndrome more than anticardiolipin antibodies and antibodies to β-2-glycoprotein I [11]. So, according to these data, antibodies to factor XII may be implicated in the pathophysiology of the hypercoagulable state in women with antiphospholipid syndrome showing RPL and their incidence in this clinical setting could be underestimated according to the data available from the Literature [18]. This hypothesis is really intriguing also in the case we described.

The reported patient affected by RPL, in fact, did not show an apparent cause of RPL other than reduction of clotting factor XII. Also laboratory tests, concerning thrombophilia, did not show common alteration of haemostasis associated to RPL such as factor V Leiden gene polymorphism or reduction of clotting inhibitors (i.e. protein C, protein S, antithrombin III). Yet, the clinical presentation and the presence of persistent prolonged aPTT suggested periodical tests concerning possible clotting factors deficiency and/or the presence of antiphospholipid syndrome. Results of this screening confirmed us the partial and acquired factor XII deficiency, due to the presence of antibodies to factor XII, in presence of transient lupus anticoagulant.

**Figure 1:**
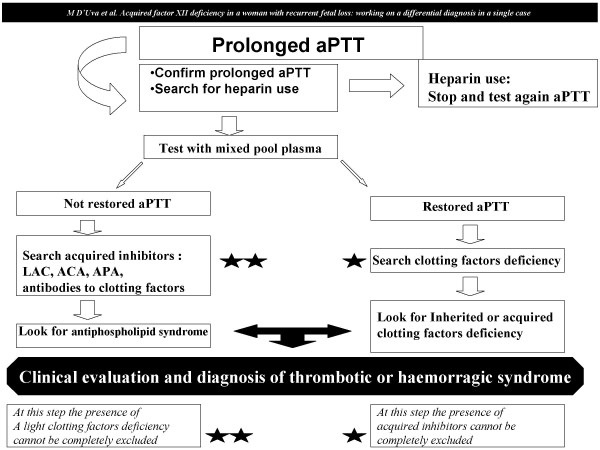
text

So, in conclusion we suggest to search in such cases also antibodies directed to clotting factors (e.g. factor XII in our case) as second step of thrombophilia screening in RPL, in particular if a persistent prolonged aPTT is present (figure 1) without an apparent cause.

## Figures and Tables

**Table 1:** Screening for disorder of haemostasis in the patient with RPL.

**Parameters (unit of measurement)**	**Results**	**Normal range**
Prothrombin time (INR)	1.15	0.8–1.2
Activated partial thromboplastin time (ratio)	1.45	0.8–1.2
Fibrinogen (mg/dL)	275	220–420
Protein C (%)	93	60–125
Protein S (%)	92	60–125
Antithrombin III (%)	104	80–120
Anticardiolipin Ab IgM (U/GPL)	1.7	< 2.0
Anticardiolipin Ab IgG (U/MPL)	3.9	< 7.0
β-2-GP I Ab	Absent	Absent
Lupus anticoagulant	Absent	Absent
Factor XII (%)	65	80–120
Factor XI (%)	113	80–120
Factor X (%)	112	80–120
Factor IX (%)	99	80–120
Factor VIII (%)	88	65–155
Factor V (%)	110	80–120
MTHFRC677T gene polymorphism	Wild type	Wild type
PTHRA20210 gene polymorphism	Wild Type	Wild type
FVL gene polymorphism	Wild Type	Wild type

INR: international normalised ratio

MTHFRC677T: methylene-tetra-hydrp-folate reductase C677T gene polymorphism

PTHRA20210G: prothrombin A20210G gene polymorphism

FVL: factor V Leiden gene polymorphism

β-2-GP I Ab: Antibodies to β-2-glycoprotein I

**Table 2:** Monitor of the alteration of haemostasis in the patient with RPL.

**Parameters (Unit of measurement)**	**First screening**	**Second screening**	**Third screening**	**Fourth screening**	**Normal value**
aPTT (ratio)	1.45	1.36	1.28	1.44	0.8–1.2
Factor XII (%)	65	50	55	43	80–120
Anticardiolipin Ab IgM (U/MPL)	1.7	1.3	1.5	1.5	< 2.0
Anticardiolipin Ab IgG (U/GPL)	3.9	5.4	3.8	2.5	< 7.0
β-2-GP I Ab	Absent	Absent	Absent	Absent	Absent
Lupus anticoagulant	Absent	Absent	Present	Absent	Absent
Anti-factor XII Ab	Not tested	Not tested	Not tested	Present	Absent
**Antinuclear antibodies (ANA)**	**Absent**	**Not tested**	**Not tested**	**Absent**	**Not tested**

aPTT: activated partial thromboplastin time

ab: antibodies

β-2-GP I Ab: Antibodies to β-2-glycoprotein I
